# Distinct PKA Signaling in Cytosolic and Mitochondrial Compartments in Electrically Paced Atrial Myocytes

**DOI:** 10.3390/cells11142261

**Published:** 2022-07-21

**Authors:** Noa Kirschner Peretz, Sofia Segal, Ido Weiser-Bitoun, Yael Yaniv

**Affiliations:** Laboratory of Bioelectric and Bioenergetic Systems, Faculty of Biomedical Engineering, Technion-IIT, Haifa 3200002, Israel; snk@campus.technion.ac.il (N.K.P.); sofi890@campus.technion.ac.il (S.S.); idow@campus.technion.ac.il (I.W.-B.)

**Keywords:** A-kinase anchoring proteins, cAMP, mitochondria, atrial myocytes

## Abstract

Protein kinase A (PKA) is a key nodal signaling molecule that regulates a wide range of cellular functions in the cytosol and mitochondria. The distribution of A-kinase anchoring proteins that tether PKA, the local interaction with degradation molecules, and regulation by Ca^2+^, may lead to distinct spatiotemporal cAMP/PKA signaling in these compartments. In this work, FRET-based sensors were used to investigate PKA signaling in the cytosol, outer mitochondrial membrane (OMM), and mitochondrial matrix (MM) and its crosstalk with Ca^2+^ in response to electrical stimulation of cultured rabbit atrial cells. A gradual decrease in PKA activity eliminating the ability of the atrial cells to respond to physiological electrical stimulation, was observed upon treatment of cells with H-89. Chelation of intracellular Ca^2+^ by BAPTA reduced PKA activity and diminished its response to forskolin, an AC stimulator. Under basal conditions, PKA activity in response to forskolin was lower in the OMM compared to the cytosol and MM. In response to electrical stimulation in the presence of ISO, distinct compartmentalization of PKA activity was observed, with higher activity in the cytosol and MM than in the OMM. Thus, distinct Ca^2+^-dependent spatiotemporal cAMP/PKA signaling exists in atrial cells, likely mediating its excitation and mitochondrial function.

## 1. Introduction

Protein kinase A (PKA) is a key nodal signaling protein that regulates a wide range of molecules in the cell, including in the mitochondria [1]. In the cell, PKA is bound to A-kinase anchoring proteins (AKAP), which tether PKA in proximity to local targets in the cytosol, the outer mitochondrial membrane (OMM), and the intermembranal space [2,3,4]. AKAP distribution, interaction with local phosphodiesterase (PDE, which degrades cAMP), and degradation by phosphatases (PP) leads to distinct spatiotemporal cAMP/PKA signaling in the cytosol and mitochondrial compartments [5,6].

In atrial cells, PDE2-4 [7] and PDE1a [8] are highly expressed. High expression of PP1c, PP2Ac [9], and PP1a [10] was also documented in atrial cells. Ca^2+^ activates PDE1a [11] and PP1a [12], either directly or through Ca^2+^/calmodulin-dependent protein kinase II (CaMKII) signaling. In atrial cells, cAMP/PKA signaling is also driven by Ca^2+^-activated adenylyl cyclase (AC) [13]. This crosstalk between Ca^2+^ and PKA signaling suggests that Ca^2+^ is a putative regulator of PKA signaling. Because Ca^2+^ cycling has distinct spatiotemporal characteristics in the cytosol and in the mitochondria [14], it may also contribute to distinct spatiotemporal cAMP/PKA signaling in these compartments.

Although PKA compartmentalization was shown in ventricular myocytes [15], its distribution in atrial cells is unknown and monitoring of live PKA dynamics in the cytosol and mitochondrial compartments has not been reported. Additionally, the extent to which Ca^2+^ regulates PKA activity in atrial cells has yet to be determined. More specifically, action-potential-induced elevation of intracellular Ca^2+^ occurs during excitation–contraction coupling. It is not known, however, if pacing atrial cells at a physiological rate (1–3 Hz) that induces elevation of intracellular Ca^2+^ can regulate PKA and whether it regulates it distinctly in the cytosol or mitochondria.

To investigate spatiotemporal PKA dynamics and its crosstalk with Ca^2+^ in atrial cells, we tested four hypotheses: (i) PKA mediates electrical pacing of atrial cells, (ii) Ca^2+^- is an important regulator of PKA activity, (iii) compartmentalization of PKA exists, and (iv) in response to electrical stimulation, distinct PKA activity exists in the cytosol, OMM, and mitochondrial matrix (MM).

Here, we targeted fluorescence resonance energy transfer (FRET)-based PKA activity sensors to the cytosol, OMM, and MM to visualize compartment-specific changes in PKA activity, in real time, in live cultured atrial cells. Our data demonstrated that the mitochondria harbors at least two distinct PKA microdomains (OMM and MM) with unique signaling characteristics that differ from the cytosolic PKA pathways.

## 2. Materials and Methods

### 2.1. Animal Use

Animals were treated in accordance with the Technion Ethics Committee. The experimental protocols were approved by the Animal Care and Use Committee of the Technion (Ethics approval: IL-001-01-19).

### 2.2. Atrial Cell Isolation

Atrial tissue was isolated as previously described [16], from healthy male white New Zealand White rabbits, weighing 2.3–2.7 kg. Each rabbit was weighed and sedated through intramuscular administration of ketamine (0.1 mL/kg) and xylazine (0.1 mL/kg). An intravenous cannula was inserted in the rabbit’s ear, after which, 200 mg/mL sodium pentobarbital diluted with heparin, were delivered through the IV cannula. The adequacy and efficiency of the anesthesia were examined by observing the loss of reflexes in the eye and foot. Cells were dispersed by gently pipetting from the atrial tissue preparation in KB solution (containing (in mM): L-glutamic acid 70, KCl 30, KH_2_PO_4_ 10, HEPES 10, taurine 20, glucose 10, MgCl_2_ 1, and EGTA 0.3 (pH 7.38 with KOH)), and then filtering the sample through a 150 μm mesh, and storing at 4 °C, for up to 6 h.

### 2.3. Culture Procedure

Immediately after isolation, cells were placed in a short-term culture (24 h), as previously described [17]. The cells were seeded onto 35 mm glass dishes (MatTek Corporation, Ashland, MA, USA) coated with 25 µg/mL laminin (Sigma Aldrich, St. Louis, MO, USA) dissolved in PBS (in PBS + 1% penicillin-streptomycin (PS), no Ca^2+^ and no Mg^2+^, Sigma Aldrich), in a serum-enriched culture medium composed of M199, 5% fetal bovine serum (FBS, Gibco), 2% PS, and 0.1% 2, 3-butanedione monoxime (BDM). BDM was washed out prior to the experiments.

### 2.4. Cell Infection with FRET Probes

Cells were infected with adenoviral particles carrying either the cytosolic, OMM, or MM PKA probe, i.e., pcDNA3-A-kinase activity receptor 4 (1 µL/dish) (pcDNA3-AKAR4, Addgene, Watertown, MA USA), yTom-AKAR4 (1 µL/dish), and 4Cox8-AKAR4 (1.75 µL/dish), respectively (OMM and MM plasmids were kind gifts from Aldebaran M. Hofer (Harvard Medical School, West Roxbury, MA, USA)). Plasmids with the FRET sensor were generated by Vector Biolabs. The cells were imaged 24 h after infection.

### 2.5. Electrical Stimulation

Electrical fields (1, 2, and 3 Hz) were created using a pair of platinum electrodes (0.008″ bare wire, A–M Systems) glued to a custom-made chamber top (see Figure S1A for an image). The wires were connected to a stimulator (Multichannel system, Reutlingen, Germany).

### 2.6. FRET Measurements

Culture medium was aspirated prior to imaging. The dishes were washed several times with HEPES solution, containing (in mM): NaCl 140, KCl 5.4, HEPES 5, glucose 10, MgCl_2_ 2, CaCl_2_ 1 (pH 7.4 with NaOH). Cells that contracted in response to electrical stimulation (which has no effect on PKA activity and which robustly expressed the reporter were used for imaging. Real-time FRET imaging experiments were performed on an LSM880 confocal microscope (Zeiss, Oberkochen, Germany) at 37 ± 0.5 °C, under a 40×/1.2 N.A water immersion lens. The cells were excited at a wavelength of 405 nm, and fluorescence emission of cyan fluorescent protein (CFP) and yellow fluorescent protein (YFP) were measured at wavelengths of 463–512 and 517–585 nm, respectively, with a 458/514 nm mirror beam splitter. Images were acquired every 1.27 s. Fluorescence images were background-corrected by subtracting the fluorescence intensity of the background with no cells from the emission intensities measured from cells expressing fluorescent reporters. The FRET ratio, yellow (FRET) intensity/cyan intensity, was then calculated. The FRET ratio of all time courses was normalized by dividing each value by the average basal value before stimulant/inhibitor addition (untreated conditions) and calculated at steady state (the value of a 10 s sliding window lowpass filter was compared to a threshold taken as the mean value of the segment starting from 30 s after the start of the segment (this part of the segment was considered transient-free). When the sliding window average was within 5% of the threshold, the transient period was considered over, and the rest of the segment was used, eliminating the values above 5%). No drift was observed in response to time-control experiments (Figure S1B, 1.007 ± 0.01 drift, *n* = 9, 3 rabbits).

### 2.7. Confocal Imaging

Atrial cells were cultured and infected with sensors as described above and incubated for 24 h. Thereafter, the cells were loaded with 10 nM MitoTracker red (for 4Cox8-AKAR4) or 25 nM MitoTracker green (for yTom-AKAR4) for 15 or 30 min, respectively, at 37 °C. Cells were bathed with the above HEPES solution at room temperature and imaged on an LSM880 confocal microscope (Zeiss) under a 63×/1.4 N.A. oil immersion objective. Figure S2A shows a confocal image of an atrial cell expressing 4Cox8-AKAR4 and loaded with MitoTracker red and demonstrates proper localization of 4Cox8-AKAR4. Figure S2B shows a confocal image of an atrial cell expressing yTom-AKAR4 and loaded with MitoTracker green, and shows proper localization of yTom-AKAR4

### 2.8. Ca^2+^ Measurements

Ca^2+^ cycling into and out of the cytosol was measured by loading atrial cells with 5 μM Fluo-4 AM (ThermoFisher Scientific) for 20 min at room temperature, and subsequently washing them with HEPES solution at 37 ± 0.5 °C. Fluorescence was imaged with a LSM880 confocal microscope (Zeiss) using a 40×/1.2 N.A. water immersion lens. Cells were excited with a 488 nm argon laser line and emission was collected with LP 505 nm, with the pinhole set to form an image of no more than a 3 μm optical slice (512 × 1 pixels at 54.35 pixels/µm and 5.56 ms/line for 1 Hz stimulation rate, and at 58.26 pixels/µm and 5.88 ms/line for 3 Hz stimulation rate). All images were recorded using the line scan function oriented to scan along the long axis of the cell, close to the sarcolemmal membrane. Ca^2+^ images were analyzed on a custom-made guided user interface (GUI) programmed in Matlab using the same parameters described in [16].

### 2.9. Drugs

PKA inhibitor (H-89), isoproterenol (ISO), AC activator (Forskolin), Ca^2+^ chelator (BAPTA-AM), 3-isobutyl-1-methylxanthine (IBMX), and all other chemicals were purchased from Sigma-Aldrich.

### 2.10. Statistics

Data are presented as mean ± SEM. ANOVA was used with the Sidak multiple comparison to differentiate when several conditions were tested. Differences were considered statistically significant at *p* ≤ 0.05. *n* is equal to the number of cells. For each protocol, at least 3 rabbits were used.

## 3. Results

### 3.1. PKA Activity Mediates the Ability of Atrial Cells to Be Electrically Paced

**Figure 1:**
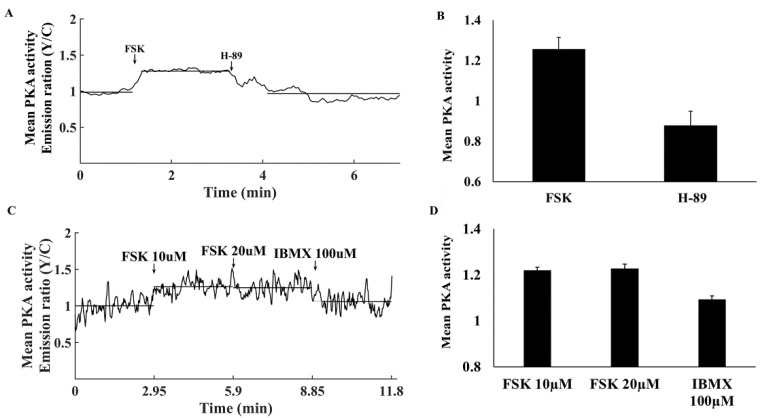
Mean PKA activity in the cytosol. (**A**) Representative example of cytosolic PKA activity (AKAR4 emission Y/C) in a single atrial cell in response to adenylyl cyclase (AC) activation by 10 µM forskolin (FSK) followed by 6 µM H-89. (**B**) Mean PKA activity in the cytosol in response to FSK and H-89 (*n* = 7, 3 rabbits). (**C**) Representative example of cytosolic PKA activity (AKAR4 emission Y/C) in a single atrial cell in response to AC activation by 10 and 20 µM forskolin (FSK) and 100 µM 3-isobutyl-1-methylxanthine (IBMX). (**D**) Mean PKA activity in the cytosol in response to FSK and IBMX (*n* = 5. 3 rabbits). Data are presented as mean ± SEM.

Characterization of basal PKA activity in the cytosol showed that it increased upon addition of the AC activator forskolin (FSK) and decreased on administration of the PKA inhibitor H-89 (Figure 1A). FSK increased PKA activity to 1.26 ± 0.06 and H-89 decreased it to 0.88 ± 0.07 (Figure 1B).

**Figure 2:**
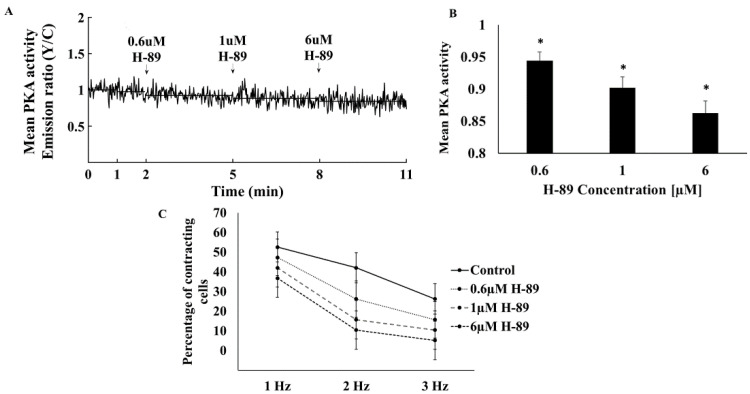
The effect of a PKA inhibitor on mean PKA activity in the cytosol and the ability to be electrically stimulated. (**A**) Representative example of cytosolic PKA activity (AKAR4 emission Y/C) in a single atrial cell in response to increasing concentrations of H-89 (0.6, 1, 6 µM). (**B**) Mean PKA activity in the cytosol in response to increasing concentrations of H-89 (*n* = 13, 4 rabbits). (**C**) Percentage of atrial cell contraction at 1, 2, and 3 Hz, in the absence vs. presence of H-89 (0.6, 1, 6 µM) (*n* = 19, 3 rabbits). Data are presented as mean ± SEM. * *p* < 0.05 compared to baseline.

To determine the relative baseline PKA activity, we compared it to its maximal and minimal values. Maximal PKA activity was achieved in response to 10 µM FSK (Figure 1C,D). Neither a higher concentration of FSK nor 100 µM IBMX further increased PKA activity. The PKA signal decreased to its minimal activity upon treatment with 6 µM H-89 (Figure 2A). At higher concentrations, atrial cells could not be electrically stimulated and the majority did not survive. PKA activity was 31 ± 10% when normalizing it to the activities measured in response to FSK and H-89.

To test the relationship between PKA activity and the ability of the cells to be electrically stimulated, cells were treated with increasing concentrations of H-89. A gradual increase in the H-89 concentration in the range of 0.6–6 µM markedly reduced PKA activity (Figure 2A,B), in parallel to a reduction in the ability of atrial cells to be electrically stimulated at all frequencies between 1 and 3 Hz, where 3 Hz is the spontaneous beating rate of sinoatrial node cells [18] (Figure 2C).

### 3.2. Ca^2+^-Activated AC Is an Important Regulator of PKA Activity in Atrial Cells

**Figure 3:**
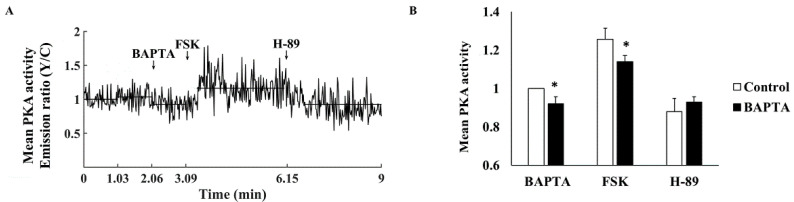
The effect of a Ca^2+^ chelator on mean PKA activity in the cytosol. (**A**) Representative example of cytosolic PKA activity (AKAR4 emission Y/C) in a single atrial cell treated with BAPTA-AM (10 µM). (**B**) Mean PKA activity in the cytosol of atrial cells treated with BAPTA-AM followed by 10 µM forskolin (FSK) and 6 µM H-89 (*n* = 16, 5 rabbits). Data are presented as mean ± SEM. * *p* < 0.05 compared to control conditions.

To determine whether Ca^2+^ regulates PKA activity, cells were treated with BAPTA to chelate cytosolic Ca^2+^. Figure 3A shows a representative example of the effects of BAPTA on PKA activity. BAPTA reduced PKA activity by 9.6 ± 2.9% compared to control (Figure 3B). To verify that reduction in Ca^2+^ limits the maximal PKA activity generated by activation of AC, we stimulated PKA activity with FSK. Coadministration of BAPTA and 10 µM FSK resulted in reduced PKA activity as compared to the maximal PKA activity generated by activation of AC by FSK (1.14 ± 0.03 compared to 1.26 ± 0.06 in control) (Figure 3B), suggesting that Ca^2+^ indeed regulates PKA activity. In contrast, no change was documented upon treatment with both BAPTA and H-89 (0.93 ± 0.03 compared to 0.88 ± 0.07 in control).

### 3.3. PKA Compartmentalization in the Cytosol, MM, and OMM

**Figure 4:**
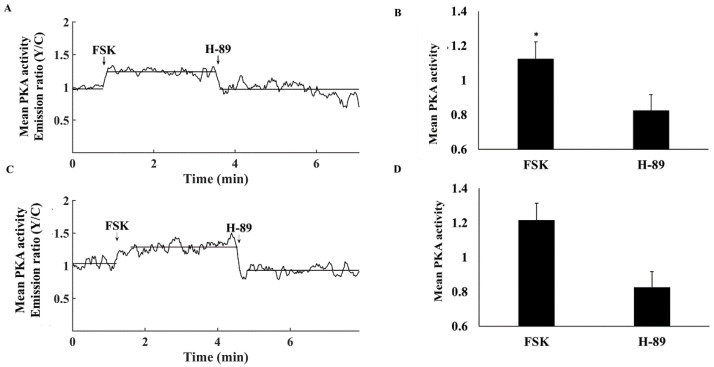
Mean PKA activity in the mitochondrial compartments. (**A**) Representative example of PKA activity in the outer mitochondrial membrane (OMM-AKAR4 emission Y/C) in response to adenylyl cyclase (AC) activation by 10 µM forskolin (FSK) and 6 µM H-89. (**B**) Mean PKA activity in the OMM in response to FSK and H-89 (*n* = 7, 3 rabbits). (**C**) Representative example of mitochondrial matrix PKA activity (MM-AKAR4 emission Y/C) in response to AC activation by 10 µM forskolin (FSK) and 6 µM H-89. (**D**) Mean PKA activity in MM in response to FSK and H-89 (*n* = 12, 3 rabbits) and 6 µM H-89. Data are presented as mean ± SEM. * *p* < 0.05 compared to cytosol.

To verify that PKA penetrates the MM as was documented in other tissues [6], we used sensors targeted to the OMM or to MM. Figure 4A shows a representative example of the effects of FSK and H-89 on PKA activity in the OMM. FSK increased PKA activity in the OMM to 1.12 ± 0.06 and H-89 decreased it to 0.83 ± 0.09 (Figure 4A,B). The FSK-associated increase in PKA activity was lower in the OMM than in the cytosol, while responses to H-89 were identical in the cytosolic vs. OMM. PKA activity was 26 ± 7% when normalizing to the values in response to FSK and H-89. Figure 4C shows a representative example of the effects of FSK and H-89 on PKA activity in MM. Similarly, FSK increased PKA activity in the MM to 1.26 ± 0.04, while H-89 decreased it to 0.93 ± 0.03 (Figure 4C,D). While values aligned with those obtained for cytosolic PKA, the FSK-associated increase in PKA activity was lower in the OMM than in the MM. Both had the same response to H-89. PKA activity was 24 ± 5% when normalizing to the values in response to FSK and H-89.

### 3.4. Electrical Stimulation Elicits Distinct PKA Activity in the Cytosol

**Figure 5:**
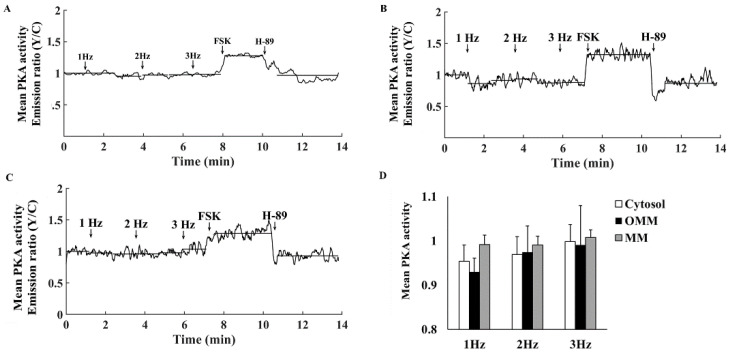
PKA activity in response to electrical stimulation. Representative example of (**A**) cytosolic PKA activity (AKAR4 emission Y/C), (**B**) outer mitochondrial membrane PKA activity (OMM-AKAR4 emission Y/C), and (**C**) mitochondrial matrix PKA activity (MM-AKAR4 emission Y/C) in response to electrical stimulation (1, 2, or 3 Hz) followed by adenylyl cyclase (AC) activation by 10 µM forskolin (FSK) and PKA inhibition by 6 µM H-89. (**D**) Mean PKA activity in the cytosol (*n* = 7, 3 rabbits), outer mitochondrial membrane (*n* = 7, 3 rabbits), and mitochondrial matrix (*n* = 12, 3 rabbits) in response to electrical stimulation (1, 2, or 3 Hz) followed by 10 µM FSK and 6 µM H-89. Data are presented as mean ± SEM.

To determine whether electrical stimulation can change PKA activity, PKA activity was measured in cells electrically stimulated at a rate of 1–3 Hz. Figure 5A–C show representative examples of the effects of increased electrical stimulation rate on PKA activity in the cytosol, OMM, and MM, respectively. On average, PKA activity in either compartment did not change in response to electrical stimulation (Figure 5D).

**Figure 6:**
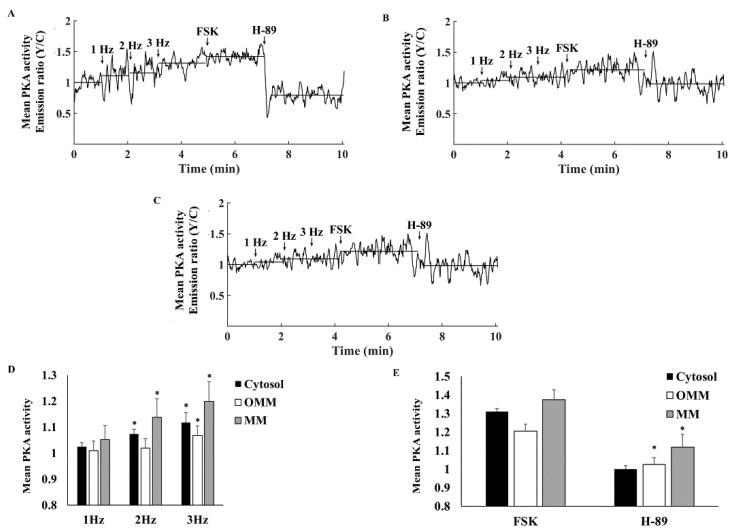
Mean PKA activity in response to electrical stimulation in the presence of isoproterenol. Representative example of (**A**) cytosolic PKA activity (AKAR4 emission Y/C), (**B**) outer mitochondrial membrane PKA activity (OMM-AKAR4 emission Y/C), and (**C**) mitochondrial matrix PKA activity (MM-AKAR4 emission Y/C) in response to electrical stimulation (1, 2 or 3 Hz) followed by adenylyl cyclase (AC) activation by 10 µM forskolin (FSK) and 6 µM PKA inhibition by H-89. (**D**) Mean PKA activity in the cytosol (*n* = 10, 3 rabbits), outer mitochondrial membrane (*n* = 7, 3 rabbits), and mitochondrial matrix (*n* = 7, 3 rabbits) in response to electrical stimulation (1, 2 or 3 Hz) (**E**) followed by 10 µM FSK and 6 µM H-89. Data are presented as mean ± SEM. * *p* < 0.05 compared to 1 Hz.

**Figure 7:**
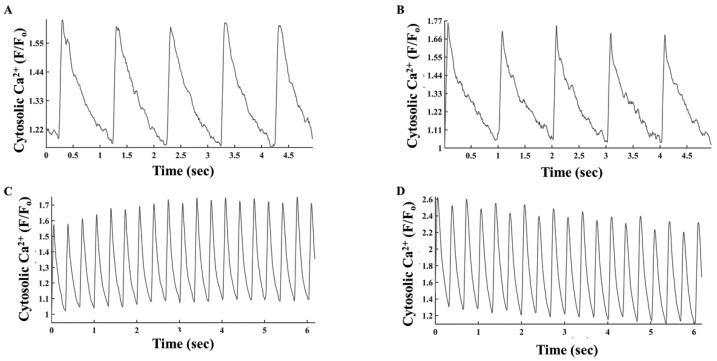
Representative example of cytosolic Ca^2+^ levels (**A**) in the absence and (**B**) presence of isoproterenol and electrical stimulation at 1 Hz. Representative example of cytosolic Ca^2+^ levels (**C**) in the absence and (**D**) presence of isoproterenol and electrical stimulation at 3 Hz.

When the heart rate increases, brain receptors are stimulated, activating AC-cAMP/PKA signaling, which enables the targeted mechanisms to adapt to a higher beating rate [19]. Thus, we explored if electrical stimulation in the presence of isoproterenol (ISO), which activates AC-cAMP/PKA signaling, changes PKA activity. Figure 6A–C show representative examples of the effects of increased electrical stimulation rates in the presence of ISO on PKA activity in the cytosol, OMM and MM, respectively. In the cytosol, electrical stimulation (2 and 3 Hz) in the presence of ISO significantly increased PKA activity (Figure 6D). Similar trends were documented for PKA activity in MM. However, in the OMM, PKA activity in the presence of ISO only increased significantly in cells subjected to a stimulation rate of 3 Hz. Overall, in the presence of ISO and under an electrical stimulation rate of 3 Hz, PKA activity increased by 19.9 ± 7%, 11.7 ± 4%, and 6.7 ± 4% from its levels measured at a 1 Hz stimulation rate in the MM, cytosol, and OMM compartments, respectively. Thus, the changes in PKA activity when increasing the stimulation rate from 1 to 3 Hz were significantly different in the cytosol compared to the OMM compartment. The presence of ISO did not affect the PKA response to FSK in either compartment. However, the magnitude of reduction in PKA activity after addition of ISO and H-89 was lower in the OMM and MM (Figure 6E) as compared to their respective levels without ISO (Figure 4). In parallel, administration of ISO and stimulation with 1 or 3 Hz increased the cytosolic Ca^2+^ amplitude by 52 ± 20% (*n* = 6) and 36 ± 14% (*n* = 4), respectively (Figure 7). These results suggest that only after ISO activates cascades that increase cytosolic Ca^2+^ to a certain level, is PKA activated by Ca^2+^ during electrical stimulation.

## 4. Discussion

**Figure 8:**
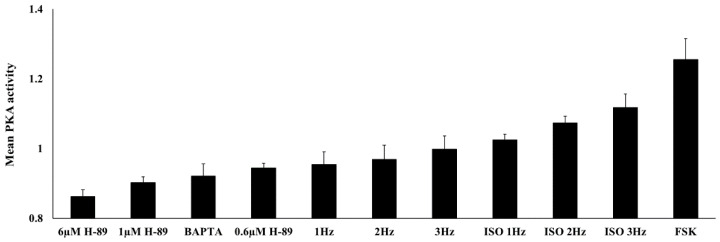
Summary of PKA activity in the cytosol under different interventions (electrical stimulation and drug perturbations). ISO—isoproterenol; FSK—forskolin. Data are presented as mean ± SEM.

The present study investigated PKA signaling compartmentalization in atrial cells in response to electrical stimulation. For the first time, live PKA dynamics was measured in electrically stimulated atrial cells in basal state and in response to drug perturbations (Figure 8). Drugs that led to a reduction in PKA activity (Figure 1) reduced the ability of atrial cells to be electrically stimulated. In addition, reductions in Ca^2+^ levels (Figure 3) by a Ca^2+^ chelator or increases in Ca^2+^ levels by application of ISO (Figure 6), affected PKA activity, suggesting that Ca^2+^ is an important regulator of PKA activity. Finally, in response to a PKA activator (Figure 2 and Figure 4) or electrical stimulation in the presence of ISO (Figure 6), distinct compartmentalization of PKA activity was observed, with higher activity in the cytosol and MM than in the OMM.

We showed here that maintenance of PKA activity is important for regulation of atrial function; reductions in PKA activity eliminated the ability of atrial cells to be electrically stimulated. PKA regulates a wide range of cellular processes, including ion channel kinetics, Ca^2+^ release and uptake, contraction and energetic balance [1,20]. In response to PKA inhibition, a reduction in L-type current was documented in human atrial cells [21], ISO enhancement was prevented in canine atrial cells [22], and acetylcholine elicitation of cAMP-mediated stimulation of Ca^2+^ was prevented in cat atrial myocytes [23]. Therefore, it is not surprising that reductions in PKA activity downregulated excitation contraction coupling of atrial cells.

In addition, this work showed that Ca^2+^ is an important regulator of PKA activity. Ca^2+^-activated AC in atrial cells [13] has been shown to stimulate the cAMP-dependent PKA cascade. In turn, PKA-enhanced Ca^2+^ releases from L-type channels and releases and uptake of Ca^2+^ from and into the sarcoplasmic reticulum (SR) increase. Consequently, positive feedback on AC activity is elicited. On the other hand, the Ca^2+^-dependent activation of PDE1a, which is highly expressed in atrial cells [8]. Moreover, PP11a, which is activated by Ca^2+^ [10], is also expressed in atrial cells [9]. Thus, Ca^2+^ also downregulates the cAMP-dependent PKA cascade, resulting in negative feedback on AC activity. The overall level of PKA activity is determined by the balance between the two afferent mechanisms and overall Ca^2+^ levels. The low basal PKA activity (Figure 8) was brought to close to 50% of its maximal activation in the presence of ISO (assuming full activation with FSK and zero activation with 6 µM H-89). Thus, electrical stimulation in the presence of ISO can change PKA around this working point. ISO increased Ca^2+^ levels, and only under these conditions was cell sensitivity to electrical stimulation significant.

Compartmentalization of PKA activity in response to afferent activators FSK and electrical stimulation, manifested by lower levels in the OMM as compared to the cytosol and MM. This may be dictated by different distributions of A-kinase anchoring proteins and PDEs. The similar responses of MM and cytosolic PKA suggest that PKA activity may mediate ATP supply-to-demand matching, through phosphorylation of several mitochondrial proteins, complexes I–V, and voltage-dependent anion channels [24,25,26]. Note, that because Ca^2+^ is an important regulator of PKA activity, increases in mitochondrial Ca^2+^ in response to electrical stimulation can upregulate PKA activity and consequently, ATP supply-to-demand can be indirectly regulated by Ca^2+^. Further evidence is needed to prove this hypothesis.

### Study Limitation

We measured PKA in cultured cells expressing PKA probes and not in freshly isolated cells. However, using our method, atrial cells maintained bioelectric, biophysical, and bioenergetic function [17]. Thus, the PKA activity in cultured and fresh atrial cells is presumably similar. 

Inhibition of PKA by H-89 did not reduce PKA activity to zero. Higher concentrations of H-89 had an indirect effect [27] and killed the atrial cell which led to an abrupt increase in PKA, activity accompanied by a decline that related to probe release.

The observed difference in FRET sensor signal intensities in the different compartments may be the result of differential phosphatase activities, sensor expression levels, or other factors associated with the sensor, rather than PKA activity. However, the similar effect noted in HeLa cells [6] and the different PKA activity of the sensors in the different cell compartments (in response to electrical stimulation in the presence of ISO or in response to FSK) imply that rather the phosphatase and PDE distribution drove the effect.

AKAP tethers PKA in proximity to local targets in the cytosol, the OMM, and the intermembranal space. Disruption of AKAP activity may lead to a reduction in the PKA signal. Because different AKAPs anchor PKA at different locations, disturbing specific AKAPs can change PKA activities only at certain locations. Future experiments are needed to prove this theory.

## Data Availability

Data is available upon request.

## Supplementary Materials

The following are available online at https://www.mdpi.com/article/10.3390/cells11142261/s1; Figure S1: (A) Custom-made chamber for electrical stimulation. (B) Time control of mean PKA activity. Figure S2: Confocal images of atrial cells expressing (A) 4Cox8-AKAR4 (mitochondrial membrane) with MitoTracker red or (B) yTom-AKAR4 (outer mitochondrial membrane) with MitoTracker green, or (C) 4Cox8-AKAR4 (mitochondrial membrane) alone or (D) yTom-AKAR4 (outer mitochondrial membrane) alone, suggesting proper localization of the probes. Movie S1. Representative movie (example in Figure 5C) of mitochondrial matrix PKA activity (MM-AKAR4 emission Y/C) in response to electrical stimulation (1, 2, or 3 Hz), followed by adenylyl cyclase (AC) activation by 10 µM forskolin (FSK) and PKA inhibition by 6 µM H-89.
