# Supplementary figures and images for: Distinct PKA Signaling in Cytosolic and Mitochondrial Compartments in Electrically Paced Atrial Myocytes
Source: Cells. 2022 Jul 21;11(14):2261. doi: 10.3390/cells11142261 (PMC9319046; doi:10.3390/cells11142261)

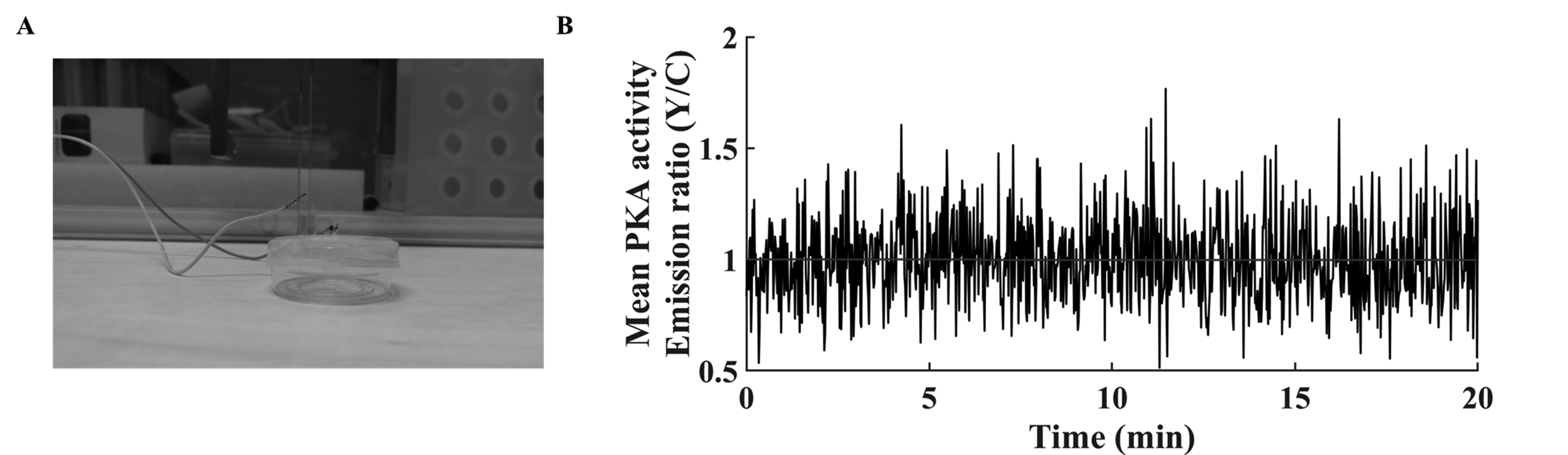

Supplement: Supplementary file 1 [file cells-11-02261-s001.zip › Figure_S1.tif]

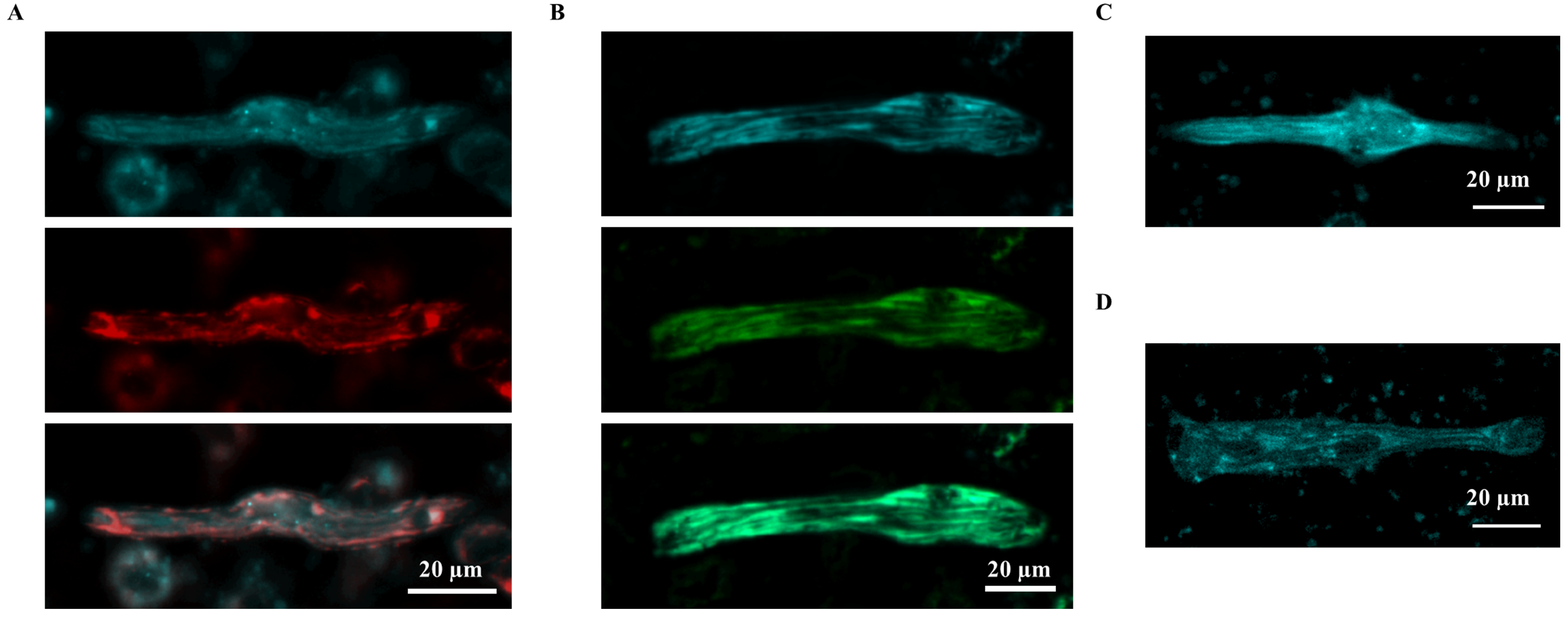

Supplement: Supplementary file 1 [file cells-11-02261-s001.zip › ‏Figure_S2.tif]
